# Influence of Synthetic Limestone Sand on the Frost Resistance of Magnesium Potassium Phosphate Cement Mortar

**DOI:** 10.3390/ma15196517

**Published:** 2022-09-20

**Authors:** Qianqian Wu, Yuying Hou, Jiangtao Mei, Jianming Yang, Tao Gan

**Affiliations:** 1 Department of Civil Engineering, Sanjiang University, Nanjing 210012, China; 2 China Construction Eighth Bureau Third Construction Co., Ltd., Nanjing 210012, China; 3 Nanchang Municipal Construction Group Co., Ltd., Nanchang 330013, China

**Keywords:** synthetic limestone sand, magnesium potassium phosphate cement mortar, freezing–thawing cycles, frost resistance, flexural and compressive strength

## Abstract

Synthetic limestone sand has advantages, such as stable quality and adjustable particle size distribution, and has gradually substituted high-quality natural sand as a fine aggregate in concrete production. The project team has prepared Magnesium Potassium Phosphate Cement (MKPC) mortar by replacing part of the river sand with machine-made limestone sand in equal amounts, which proves that its physical and mechanical properties are obviously better than mortar prepared by whole river sand. However, the research on the impact of machine-made limestone sand on the durability of MKPC mortar has not been carried out. As the repairing material of concrete structures, the frost resistance of MKPC mortar must be evaluated. In this study, the effect of synthetic limestone sand on the frost resistance of Magnesium Potassium Phosphate Cement (MKPC) mortar was investigated by characterizing the strength, mass loss rate, and water absorption of specimens subjected to freeze–thaw cycling. MKPC mortars prepared using solely river sand (M0) or limestone sand (M1) were completely degraded after 225 freezing–thawing cycles in water, whereas the flexural and compressive strengths of MKPC mortar (M2) prepared using both river and synthetic limestone sands was 29.3 and 22.0% of the initial strengths, respectively. The water freeze–thaw resistance of M2 specimens were significantly higher than that of M0 and M1 specimens, and the sulfate freeze–thaw resistance of M1 and M2 were significantly higher than that of M0. The mass loss of MKPC mortar is not more than 0.4% when it is frozen and thawed 225 times in water and 5% Na_2_SO_4_ solution, which is far lower than the damage standard of 5%. Based on the favorable composition of the two aggregates, the initial open porosity of M2 was relatively low, owing to the lower water–cement ratio of the mortar at the same flow rate.

## 1. Introduction

Reinforcement corrosion, freeze–thaw damage in cold regions, and physicochemical effects of harsh environments are recognized as the main causes of concrete durability damage [1]. Freeze–thaw damage to concrete, such as various marine and hydraulic buildings, and bridges and pavements often occurs in cold areas that experience regular rainwater [2]. These conditions severely affect the normal performance of buildings and their safety for long-term use. Similarly, the deterioration of concrete performance caused by salt erosion is also severe, threatening the safety of a large number of concrete structures in the western part of China and the coastal and offshore regions [3]. Among the various types of concrete salt erosion, sulfate erosion damage is the most common and is extremely harmful to the concrete material. Moreover, a combination of sulfate erosion and the dual effect of freezing and thawing complicates the damage to fabricated concrete structures [4,5].

Magnesium phosphate cement(MPC) is an inorganic cementitious material with phosphate as the binding phase produced by the acid–base chemical reaction of alkali metal magnesium oxide, a soluble phosphate, and additives in a specific ratio [6]. MPC has advantages such as high early strength, good volume stability, good compatibility with silicate cement-based materials, and good durability [6,7,8,9,10]. Therefore, MPC has been promoted and applied in the field of concrete structure repair and reinforcement [7,9]. When MPC is applied as a concrete repair material, its durability under harsh service conditions should be considered.

Some experimental studies on MPC resistance to freezing and salt corrosion have been reported thus far [10,11,12]. Yang et al. [10] evaluated the resistance of MPC to salt freezing and spalling by immersing one side of MPC-based specimens in 3% Sodium chloride (NaCl) solution and subjecting MPC specimens to freezing and thawing cycles of 3 h at (−20 ± 2) °C and 3 h at (20 ± 5) °C, respectively. MPC-based materials were confirmed to have higher resistance to salt freezing and spalling compared with non-air-entrained conventional silicate cement concrete. Ding et al. [11] subjected MPC and conventional silicate cement mortar specimens to 16 h of freezing and 8 h of thawing in a 4% calcium chloride (CaCl_2_) solution and confirmed that the surface of the conventional silicate cement mortar specimens were severely spalled after 30 freezing–thawing cycles, whereas that of MPC mortar specimens were flat. Based on structural microscopic analysis, it was concluded that the good resistance of MPC mortar to salt and frost spalling is owing to its low water saturation, void ratio of the hardened slurry, and uniform distribution of closed pores, which provide a buffer space for the condensed water molecules during freezing and reduce the internal expansion pressure of the cement. Yue Li et al. [13] investigated the strength development and microstructural evolution of the MPC net slurry and mortar in water, and NaCl and Sodium sulfate (Na_2_SO_4_) solutions. Water has the greatest impact on strength, NaCl solution has the second greatest, and Na_2_SO_4_ solution has the smallest impact on strength. The addition of quartz sand had a negative effect on the density and strength loss of the MPC microstructure, whereas fly ash had a positive effect on these properties. The evaluation of the strength and volume deformation of MPC net slurry specimens immersed in freshwater, seawater, and 5% Na_2_SO_4_ solutions for an extended period was conducted, which confirmed that the MPC net slurry has good resistance to water, seawater, and sulfate attacks, and fly ash further improved the corrosion resistance of MPC mortar [14,15,16]. However, regarding the salt freezing resistance of the MPC system, reported studies have used NaCl solution as the heat transfer medium and confirmed that the MPC system has a good resistance to sodium chloride freezing and spalling [12,14]. In addition to chloride salts, sulfates, especially Na_2_SO_4_, are also prevalent in the typical MCP working environments such as marine, salt lake, and groundwater. The strength development, volume deformation, and mass loss of an MPC net slurry subjected to freezing–thawing cycles in water, and 3.5% NaCl and 5% Na_2_SO_4_ solutions were investigated in the early stage, and it was confirmed that the performance of MPC net slurry specimens in 5% Na_2_SO_4_ solution was the most severely degraded. However, the addition of appropriate amounts of limestone powder and silica fume significantly improved the sulfate freeze–thaw resistance of MPC mortar [17]. Unfortunately, considering that most MPC-based materials used for concrete structure repair are MPC mortar or MPC concrete containing aggregate, until now, no research has been done on the frost resistance and sulfate freeze–thaw resistance of MPC paste containing aggregate.

Owing to the scarcity of high-quality natural sand resources, synthetic sand has become an ideal alternative. Synthetic limestone sand has advantages such as stable quality and adjustable particle size distribution and has been widely used as a fine aggregate in cement concrete production [18]. In this study, magnesium potassium phosphate cement (MKPC) mortar was prepared by substituting river sand with synthetic limestone sand at a 1 to 1 ratio, and the physical and mechanical properties of the resultant mortar were characterized and compared with those of mortar prepared using solely river sand. The physical and mechanical properties of MKPC mortar were significantly better than those of MKPC mortar prepared using solely river sand. Notably, the type of aggregate species has a significant effect on the interfacial behavior with the MKPC hardened body [19]. Furthermore, the water corrosion behavior of MKPC mortar differs from that of the MKPC net slurry because of the varying interfacial effects of different aggregates [13]. Therefore, this study also aims to investigate the effect of synthetic limestone sand on the sulfate freeze–thaw resistance of MKPC mortar by evaluating and comparing the physical and mechanical properties of MKPC mortar specimens before and after the process of sulfate freeze–thaw cycling, and to investigate the mechanism of this effect, so as to provide the basis and premise for the application of MKPC mortar to the repair and reinforcement of various marine and hydraulic structures in cold regions.

## 2. Materials and Methods

### 2.1. Materials

Refired magnesium oxide (MgO) powder with a specific surface area of 230 m^2^·kg^−1^ was used in this study. The MgO content in the refired magnesium oxide powder was greater than 90%, and the SiO_2_ and CaO contents were greater than 3%, as determined by X-ray fluorescence (XRF) spectroscopy. The dead-burned magnesia powder was produced by the magnesia factory of Hengren Dongfanghong Hydropower Station in Liaoning Province. The industrial-grade potassium dihydrogen phosphate (KH_2_PO_4_) was provided by Lianyungang Geli Chemical Co., Ltd. (Lianyungang, China), which had white columnar crystals with a mean particle size of 40/350~60/245 (mesh/m). The main composition of the composite inhibitor was industrial-grade borax and dodecahydrate dihydrogen phosphate [17]. The raw materials were all of industrial purity and were provided by Liaoning Kuandian chemical plant. The fine aggregates were common river sand and synthetic limestone sand, provided by Jiangsu Youchi New Material Co., Ltd. (Xuzhou, China), and the physical properties of these aggregates are listed in Table 1. The water used in the experiments was tap water. The sodium sulfate used to prepare the corrosion solution was of an analytical grade.

### 2.2. Specimen Preparation

The acid-base mass ratio of MKPC was selected to be 1.5. The compound inhibitor was added at 8 wt% relative to MKPC mortar, and the bone glue to MKPC mortar mass ratio was 1.5. The water–cement ratio of the three mortars was adjusted to ensure the same flow rate. Notably, the water–cement ratio of M2 was significantly lower than that of M0 and M1, indicating that mixing the two fine aggregates using an appropriate composition results is a more suitable combination of the different grain sizes.

Reference ASTM C348-2008 “Standard Test Method for Flexural Strength of Hydraulic-Cement Mortars”, ASTMC 349-2008 “Standard Test Method for Compressive Strength of Hydraulic-Cement Mortars (Using Portions of Prisms Broken in Flexure)” for flexural and compressive strength tests. To prepare the various MKPC specimens, the raw materials were weighed and mixed, and the MKPC mortar was poured into 40 × 40 × 160 mm^3^ molds (for flexural and compressive strength tests) and Φ50 × 150 mm^3^ molds (for mass loss and water absorption tests). After vibration, the excess slurry was removed with a scraper, and the molds were sealed with cling film and stored under ambient conditions. MKPC specimens were demolded after 5 h of hydration and maintained at a temperature of 20 ± 5 °C and humidity of 60 ± 5% until the specified age. The flexural and compressive strengths of the mortar specimens are listed in Table 2.

### 2.3. Test Methods

The freeze–thaw cycle testing of MKPC mortar specimens was performed according to the rapid freeze–thaw test method for concrete (GB/T50082-2009) [21] and mortar (JGJ/T70-2009) [22]. Zhao X et al. [23]. have studied the frost resistance of concrete, which reported that the mass loss rate, strength loss rate (including flexural, compressive, and tensile strengths), and dynamic elastic modulus loss rate of concrete can be used as an evaluation index of concrete deterioration. However, the flexural strength loss rate serves as a more suitable evaluation index of freeze–thaw durability of dense high-strength concrete, by accurately indicating the degree of concrete performance reduction during the freeze–thaw process. Therefore, in this study, the flexural and compressive strengths of MKPC mortar specimens were analyzed during the freeze–thaw process. A set of 40 × 40 × 160 mm^3^ MKPC mortar specimens (flexural strength > 10.0 MPa, compressive strength > 60 MPa), with pre-drilled holes along the central axis of the longest side of the specimen were used. After the specimens were demolded, a temperature sensor (model: TWTU-0015, company: PYRINDUS, region: Brussels, Belgium) was inserted into the pre-drilled hole and sealed with MPC slurry, which served as the central temperature control standard during the freeze–thaw process. The heat transfer medium was H_2_O or 5% Na_2_SO_4_ solution. Prior to the freeze–thaw test, the MKPC specimen was soaked in the heat transfer medium for 4 d. The maximum temperature of the center was 10 °C and the minimum temperature was −15 °C. The duration of the freezing time was 3.5 h and the thawing time was 2.5 h for the freeze–thaw procedure. Subsequently, the original medium in the sleeve was replaced with fresh water or 5% Na_2_SO_4_ solution after 25 cycles to ensure the concentration of the heat transfer medium was maintained. The strength and quality of MKPC specimens were tested regularly.

The following standard test methods were applied in this study: ASTM C348-2008 “Standard Test Method for Flexural Strength of Hydraulic-Cement Mortars”, ASTM C 349-2008 “Standard Test Method for Compressive Strength of Hydraulic-Cement Mortars (Using Portions of Prisms Broken in Flexure)”. The bending and compressive strengths of MKPC specimens (40 × 40 × 160 mm^3^) were tested with a WED-300 electronic universal testing machine (company: Wuxi Jianyi Instrument Machinery Co., Ltd., area: Wuxi, Jiangsu, China), according to the standard test method. The controlled bending and compressive loading speeds were within the range of 0.04–0.06 and 2.2–2.6 kN/s, respectively. The flexural (compressive) strength of MKPC specimens subjected to freeze–thaw cycles in water or salt solutions *n* times were compared with the initial flexural (compressive) strength of MKPC specimens (saturated surface dry strength using the same immersion medium) to obtain the residual strength rate.

A set of MKPC specimens (Φ50 × 150 mm^3^, denoted as specimens for quality testing) saturated with the selected immersion medium were weighed using a balance (accuracy = 0.01 g) before the start of the freezing–thawing process, which was taken as the initial saturated surface dry mass (*W*_0_). The saturated surface dry mass (*W_n_*) of the specimen was weighed after *n* freeze–thaw cycles, and the mass loss of MKPC after *n* freeze–thaw cycles was calculated using Equation (1) [21].

(1) $$△W_{n}=\frac{\left(W_{0}-W_{n}\right)}{W_{0}}\times 100\%$$

The water absorption of the MKPC hardened bodies was tested according to ASTM C15852013. Firstly, the saturated surface dry mass (*W_n__,wet_*) of MKPC specimens (Φ50 × 150 mm^3^) after being subjected to *n* freeze–thaw cycles was weighed using an electronic balance (accuracy = 0.01 g). Subsequently, the specimens were placed in a vacuum drying oven at a temperature of 60 °C and then cooled to room temperature. The mass of the dried MKPC specimens was weighed (*W_n__,dry_*) and the water absorption of the MKPC hardened body was calculated using Equation (2) [24].

(2) $$\rho_{n}=\frac{W_{n,wet}-W_{n,dry}}{W_{n,dry}}\times 100\%$$

MKPC specimens were obtained from the samples broken during the strength test, washed with water to remove the precipitates adhered to the surface, and soaked in anhydrous ethanol to suspend hydration. Subsequently, the specimens were dried in a vacuum drying oven at 60 °C before analysis. The specimens (with skins) were powdered and sieved using a 200-mesh sieve. The physical composition of MKPC specimens was determined by X-ray diffraction (XRD, D/max-RB type). Scanning electron microscopy coupled with energy-dispersive X-ray spectroscopy (SEM-EDS) was performed on the edge of the specimens using a QUANTA200 environmental scanning electron microscope (FEI Co., Hillsboro, OR, USA) to characterize the morphology and elemental distribution of MKPC hydration products, respectively. Thermal gravimetric analysis coupled with differential scanning calorimetry (TGA-DSC) was performed using a NETZSCH STA 409 PC/PG thermal analyzer in the temperature range of 20–700 °C at a heating rate of 10 °C/min, with nitrogen as the protective gas and α-Al_2_O_3_ as the reference material.

## 3. Results and Discussion

### 3.1. Strength Development

**Figure 1:**
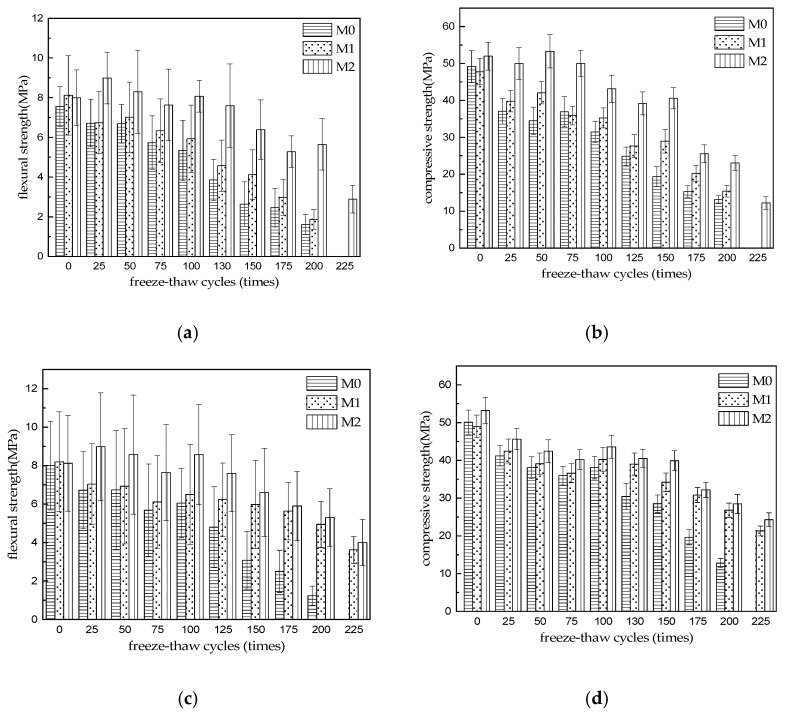
Strength development of MKPC specimens under freezing and thawing conditions in different media. (**a**) Flexural strength (in H_2_O); (**b**) Compressive strength (in H_2_O); (**c**) Flexural strength (in 5% Na_2_SO_4_); (**d**) Compressive strength (in 5% Na_2_SO_4_).

Figure 1 shows the variation in the flexural and compressive strength of MKPC mortar specimens (M0–M2) after being subjected to freeze–thaw cycles in water and sulfate solutions for different periods, where the strength of specimens at cycle number 0 is the saturated surface dry strength of MKPC specimens (at an age of 28 d) immersed in the corresponding medium. The saturated surface dry strengths of all MKPC specimens shown in Figure 1 were slightly lower than the corresponding 28 d strengths listed in Table 2, owing to the weakening of ionic bonds in the water-saturated specimens. Figure 1a,b show that the flexural and compressive strengths of the three MKPC mortar specimens gradually decreased as the number of water freeze–thaw cycles increased. The flexural and compressive strengths of M0 and M1 were greater than 75% of the initial strength values after 75 water freeze–thaw cycles, whereas the flexural and compressive strengths of M2 were greater than 75% of the initial strength values after 150 water freeze–thaw cycles. After 200 cycles, the strengths of M0 and M1 retained 21.3% (flexural) and 23.0% (compressive), and 26.6% (flexural) and 32.2% (compressive) of their initial strengths, respectively. After 225 cycles, the M0 and M1 specimens were completely degraded, whereas the flexural and compressive strengths of the M2 specimen retained 29.3 and 22.0% of their initial strength, respectively. Therefore, the freeze–thaw resistance of the M2 specimen prepared by mixing common river sand and synthetic limestone sand was significantly higher than that of MKPC mortars prepared using solely river sand (M0) and limestone sand (M1).

The flexural and compressive strengths of the three MKPC mortar specimens in Figure 1c,d decreased gradually as the number of freeze–thaw cycles in sulfate solutions increased. The flexural and compressive strengths of M0 were lower than 75% of the initial strengths after 75 freeze–thaw cycles, whereas those of M1 remained greater than 75% of the initial strengths after 125 freeze–thaw cycles, and those of M2 remained greater than 75% of the initial strengths after 150 freeze–thaw cycles in sulfate solutions. When the freeze–thaw cycling in sulfate reached 200 times, the flexural and compressive strength of M0 was 15.4 and 25.5% of the initial strengths, respectively. However, after 225 times, M0 was completely degraded, whereas the strengths of M1 and M2 were 44.1% (flexural) and 49.3% (compressive), and 43.5% (flexural) and 45.7% (compressive) of the initial strengths, respectively. Therefore, the sulfate freeze–thaw resistance of M0 was lower than its water freeze–thaw resistance. However, the sulfate freeze–thaw resistance of M1 and M2 was significantly higher than their water freeze–thaw resistance. Therefore, the sulfate freeze–thaw resistance of limestone sand-containing MKPC specimens (M1 and M2) was significantly higher than that of the MKPC mortar specimen prepared with solely river sand (M0). The improvement effect of limestone sand on the sulfate freezing and thawing resistance of the MKPC hardened body is consistent with the improvement effect of limestone powder on the sulfate freezing and thawing resistance of MKPC hardened body in literature [16].

The freeze–thaw deterioration of cement-based materials is mainly owing to the volume expansion pressure produced by the transformation of water to ice in the capillary pores, and the difference in the ice and water vapor pressures between capillaries, which results in penetration pressure. However, when subjected to rapid freezing, the hardened-body deterioration process is more appropriately described by the expansion pressure theory [19]. Under rapid freeze–thaw cycles, MKPC mortar specimens are subjected to ice expansion pressure and hydrolytic loss of MKPC [15,16,17,18,19]. As the number of freeze–thaw cycles increases, the ice expansion pressure causes a large number of cracks in the capillary walls within the MKPC hardened body and at the interface between the MKPC hardened body and the aggregate (See SEM picture for details), which results in a decrease in MKPC strength. The influence of the aggregate species on the water freeze–thaw resistance of MKPC mortar is mainly owing to the effect of the aggregate on the water–cement ratio of fresh MKPC mortar. After appropriate finer river sand and coarser limestone sand composition is achieved, the aggregate gradation is suitable, the initial water–cement ratio of fresh MKPC mortar is reduced to maintain a certain fluidity (Table 2), and the open porosity of the hardened body is reduced after 28 d of hydration (see water absorption analysis for details), and the ambient water is less likely to penetrate the hardened MKPC mortar during water freeze–thaw cycles. Therefore, the damaging effects of ice expansion pressure and hydrolytic loss on MKPC is reduced.

Under rapid freeze–thaw conditions, the structural deterioration mechanism of the MKPC mortar hardened body subjected to salt freeze–thaw corrosion was similar to that of the water freeze–thaw corroded specimens. It is known that the freezing point of the salt solution is slightly lower than that of water, and this phenomenon can reduce the ice expansion pressure generated within the hardened body during the freezing process. However, the hygroscopic nature of the salt solution increases water saturation inside the hardened body of the cementitious material and, after the supercooled water from the salt solution freezes inside the capillaries of the hardened body, it has a more damaging effect on the pore walls [25]. This caused the strength loss of the M0 specimen subjected to sulfate freeze–thaw cycling to be higher than that of the M0 specimen subjected to water freeze–thaw cycling. However, under low-temperature sulfate conditions, the sulfate anions (SO_4_^2−^) entering the hardened body combined with magnesium cations (Mg^2+^) to produce water-containing magnesium sulfate crystals, which filled the pores (see phase analysis for details). Therefore, the structure of the MKPC hardened body is dense and the corrosion medium cannot easily penetrate the structure. Notably, the reactive calcium carbonate in the hardened bodies of M1 and M2 specimens prepared using limestone sand reacts with sulfate to produce new pore-filling phases, which were not obtained by XRD analysis, owing to its low content, and are to be further investigated [16,26]. The above-mentioned effects resulted in a significantly lower strength loss in the M1 and M2 specimens under sulfate freeze–thawing conditions compared with water freeze–thawing conditions, and a significantly lower strength loss than that observed in M0 specimens under sulfate freeze–thawing conditions. The improvement effect of limestone sand on the sulfate freezing and thawing resistance of MKPC hardened body is consistent with the improvement effect of limestone powder on the sulfate freezing and thawing resistance of MKPC hardened body in literature [16].

### 3.2. Quality Loss

**Figure 2:**
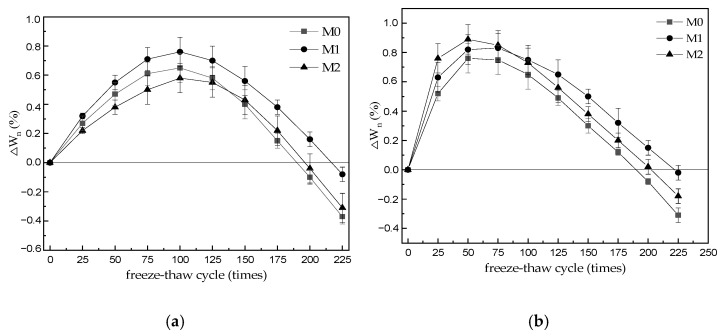
Mass change of M0–M2 specimens after a specific number of freeze–thaw cycles different varying media. (**a**) H_2_O; (**b**) 5% Na_2_SO_4_.

Figure 2 shows the mass change rate of M0–M2 specimens after a specific number of freeze–thaw cycles in H_2_O and 5% Na_2_SO_4_ solution. As shown in Figure 2a, the mass change rates of M0–M2 specimens increased as the number of freeze–thaw cycles increased, and then decreased as the number of freeze–thaw cycles increased. The mass loss of M0, M1, and M2 specimens was 0.37, 0.31, and 0.08% after 225 freeze–thaw cycles in water, respectively. As shown in Figure 2b, the mass change rates of M0–M2 specimens also increased and then decreased as the number of freeze–thaw cycles in 5% Na_2_SO_4_ solution increased. The mass loss of M0, M1, and M2 specimens was 0.31, 0.18, and 0.02% after 225 freeze–thaw cycles in the sulfate solution, respectively. Notably, under these conditions, the mass loss of M0 was slightly lower than that of M0 after 225 freeze–thaw cycles in water, whereas the mass loss of M1 and M2 was significantly lower than that of the corresponding specimens after 225 freeze–thaw cycles in water. Therefore, the mass loss of MKPC mortar was much lower than the damage criterion of 5% after 225 freeze–thaw cycles in both water and the 5% Na_2_SO_4_ solution. Moreover, these results indicate that the evaluation index of mass loss rate was insensitive to the frost resistance test of MKPC mortar under the investigated conditions. They show the mass change rate of M0–M2 specimens after a different number of freeze–thaw cycles in a 5% solution. The mass change rates of M0–M2 specimens increase with the number of freeze–thaw cycles and then decrease with the number of freeze–thaw cycles. The mass loss rates of M0, M1, and M2 specimens were 0.37%, 0.31%, and 0.08% for 225 freeze–thaw cycles of water, respectively. The mass change rates of M0–M2 specimens also tended to increase and then decrease with the increase in the number of freeze–thaw cycles of 5% solution, and the mass loss rates of M0, M1, and M2 specimens were 0.31%, 0.18%, and 0.02% for 225 freeze–thaw cycles of sulfuric acid solution, respectively, where the mass loss of M0 was slightly lower than that of M0 for 225 freeze–thaw cycles of water, and the mass loss of M1 and M2 was significantly lower than that of the corresponding specimens for 225 freeze–thaw cycles of water. The results showed that the mass loss of MKPC mortar was much lower than the damage criterion of 5% for 225 freeze–thaw cycles in both water and 5% solution, which also indicated that the evaluation index of mass loss rate was insensitive to the freezing resistance test of MKPC mortar.

**Figure 3:**
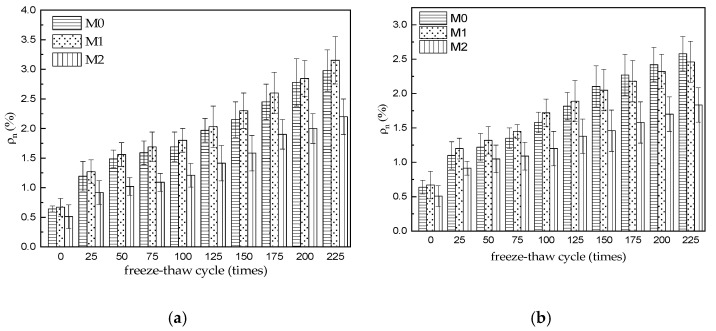
Water absorption of MKPC specimens. (**a**) Freeze–thaw cycling in H_2_O; (**b**) Freeze–thaw cycling in 5% Na_2_SO_4_.

The early hydration of fresh MKPC mortar is rapid and a large amount of heat of hydration is released [16]. The water in the slurry is consumed and evaporates owing to the concentrated release of the heat of hydration, after which the continuation of the hydration reaction is mainly dependent on the penetration of ambient water. If the MKPC hardened body is completely immersed in water, ambient water enters the open pores of MKPC, and water erosion causes the hydrolysis and loss of MgKPO_4_·6H_2_O (MKP), which is an existing hydration product in the hardened body, resulting in increased porosity and cracking in the openings of the MKPC hardened body [13,14,15,16]. MKPC mortar specimens subjected to freeze–thaw cycles were exposed to low-temperature water immersion (up to 10 °C) in the thawed state, new MKP was produced [12] and the solubility of the existing MKP hydration product decreased owing to the lower water temperature and reduced hydrolysis loss. The MKPC mortar quality increased with continued hydration at lower freeze–thaw cycles. However, as the number of freeze–thaw cycles increased, MKP loss by dissolution became a dominant factor as the ice expansion and permeability pressures of supercooled water increased the amount of open pores and cracks in the hardened body. Consequently, the quality of MKPC mortar specimens decreased owing to the spalling of the specimen surface. The M2 specimens have a denser hardened structure and lower open pore content (Figure 3), because of the lower water–cement ratio, and sustained multiple freeze–thaw cycles with less structural deterioration and a significantly lower mass loss compared with specimens M0 and M1. Under salt freeze–thaw conditions, salt crystallization and the production of new MKP caused a rapid increase in the mass of MKPC specimens during the initial stages of the freeze–thaw process, and this MKPC mass increase was higher than that of the corresponding MKPC specimens under water freeze–thaw conditions. As the number of freeze–thaw cycles increased, the structural deterioration of the hardened body increased. The loss of the MKP hydration product by dissolution, spalling of the specimen surface, and chipping of the edges resulted in a decrease in the MKPC mortar quality. In contrast, owing to salt crystallization and the production of sulfate-containing hydrates at low temperatures, the MKPC hardened body, prepared using limestone sand [26], has a denser structure and is less prone to further penetration by the sulfate corrosive medium, and thereby its final mass loss was lower than that of the corresponding specimens subjected to water freeze–thaw cycle conditions.

### 3.3. Water Absorption 

**Figure 4:**
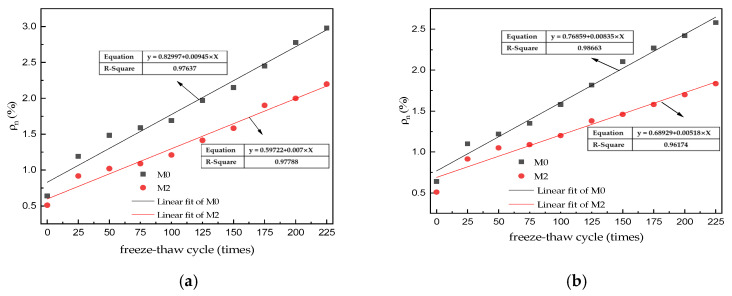
Fitting curve of water absorption of MKPC specimen. (**a**) Freeze–thaw cycling in H_2_O; (**b**) Freeze–thaw cycling in 5% Na_2_SO_4_.

The initial water absorption of the M0–M2 specimens was 0.64, 0.51, and 0.67% (before freeze–thaw cycling), respectively, as shown in Figure 3a. As the number of water freeze–thaw cycles increased, the water absorption of all three MKPC mortar specimens gradually increased. After 225 water freeze–thaw cycles, the water absorption of M0, M1, and M2 was 2.98, 3.15, and 2.20%, respectively. Figure 3b shows the water absorption of M0–M2 after freeze–thaw cycles in the 5% Na_2_SO_4_ solution. The water absorption of all three MKPC mortars increased gradually as the number of salt freeze–thaw cycles increased. The water absorption of M0, M1, and M2 specimens was 2.58%, 2.46%, and 1.83% after 225 sulfate freeze–thaw cycles, respectively, which is lower than the water absorption of the corresponding specimens after 225 water freeze–thaw cycles. Through origin fitting, it can be obtained that the water absorption of the MKPC hardened body under different solution freezing and thawing environments is linear with the number of freezing and thawing cycles (Figure 4). See Figure 4a for the linear empirical formula of freezing and thawing cycle times and water absorption rate of M0 and M2 under the water freezing and thawing environment, and see Figure 4b for the linear empirical formula of freezing and thawing cycle times and water absorption rate of M0 and M2 under the 5% sodium sulfate solution freezing and thawing environment. The correlation coefficient of the fitted empirical formula exceeds 0.97.

The open porosity of a solid material corresponds to the quality of its pore structure, and the degree of water absorption and open porosity of the same solid material are consistent with the variation of the open porosity of MKPC specimens. The initial open porosity of the M2 specimen was significantly lower than that of the M0 and M1 specimens, which is attributed to the lower water–cement ratio of the MKPC mortar with the same flow rate owing to the favorable composition of the two aggregates. As the number of freeze–thaw cycles increased, the ice expansion pressure and salt crystallization pressure in the capillary pores increased, which led to gradual deterioration of the pore structure of the hardened body.

### 3.4. Phase Analysis

**Figure 5:**
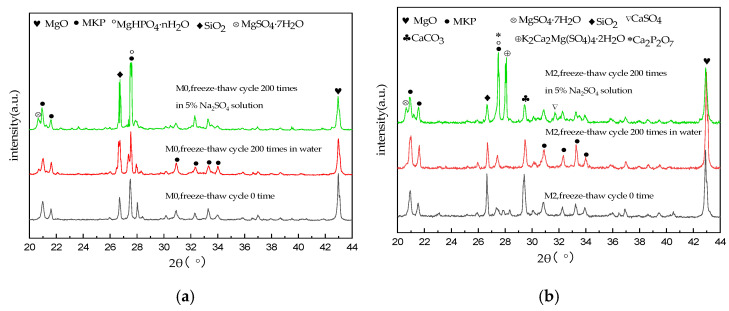
XRD diffractograms of MKPC samples (**a**) M0 and (**b**) M2.

Figure 5 shows the XRD diffractograms of M0 and M2 specimens before freeze–thaw cycling and after 200 freeze–thaw cycles in water and the sulfate solution, using the hardened body powder for XRD analysis. The characteristic peaks of MgO, MgKPO_4_·6H_2_O (MKP), MgHPO_4_·nH_2_O, and SiO_2_ were observed in the M0 specimens before freeze–thaw cycling and after 200 freeze–thaw cycles in water and sulfate, as shown in Figure 5a, and the positions of their main diffraction peaks were similar. Additionally, the main characteristic peak of MgSO_4_·7H_2_O observed in the M0 sample after 200 sulfate freeze–thaw cycles confirms that the sulfate anions (SO_4_^2−^) entered the cement matrix and reacted with magnesium cations (Mg^2+^). Figure 5b shows the XRD diffractograms of the M2 specimens. By comparing the M2 sample before the freeze–thaw cycle with the M0 sample under the same conditions (Figure 5a), an increase in the characteristic peak of CaCO_3_ was observed, which is the main component of synthetic limestone sand. Additionally, an increase in the characteristic peak of Ca_2_P_2_O_7_ was observed, which is attributed to the reaction of calcium ions on the surface of the limestone sand forming the calcium phosphate phase. For the M2 specimen obtained after 200 sulfate freeze–thaw cycles, in addition to MgSO_4_·7H_2_O, the main characteristic peaks of CaSO_4_ and K_2_Ca_2_Mg(SO_4_)_4_·2H_2_O were also observed, confirming that sulfate ions penetrated the hardened body and reacted with certain cations present in the hardened body to form these sulfate–containing mineral phases. Moreover, calcium ions in a hardened body partially dissolved and reacted, confirming that the active effect of calcium ions on the surface of limestone sand under low-temperature sulfate conditions improved the resistance of MKPC mortar specimens to sulfate freezing and thawing.

### 3.5. TG Measurements

**Figure 6:**
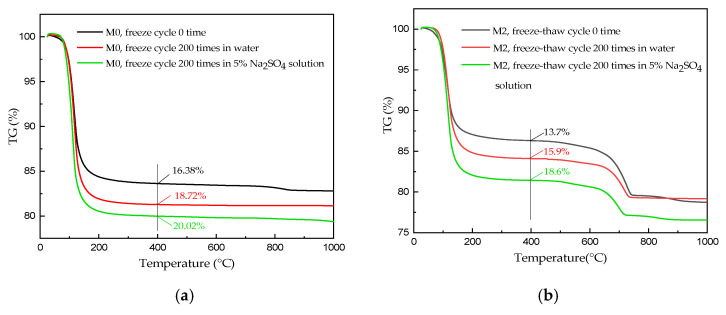
TG spectra of MKPC specimens (**a**) M0 and (**b**) M2.

Figure 6 shows the TG curves of powdered M0 and M2 samples of the broken hardened body specimens obtained before freeze–thaw cycling and after 200 cycles of freeze–thaw cycles in water and sulfate solutions. As shown in Figure 6a, the three M0 MKPC mortar samples had one distinct mass loss at approximately 100 °C, which is attributed to the loss of crystalline water of MgKPO_4_·6H_2_O crystals [16,17]. However, the mass loss of M0 subjected to varying environmental conditions at approximately 100 °C was significantly different. The weight loss of M0 samples after 200 water freeze–thaw cycles (18.72%) was significantly higher than that of M0 samples before freeze–thaw cycling (16.38%), indicating that the continued hydration of the partially reacted acid–base components dominated the MKPC mortar under these freeze–thaw conditions, which resulted in the increased MKP content in the hardened body of MKPC mortar. Moreover, the weight loss of M0 samples after 200 freeze–thaw cycles in sodium sulfate solution (20.02%) was significantly higher than that of M0 samples after 200 freeze–thaw cycles in water (18.72%), indicating a further increase in the MKP content. This is attributed to the slight increase in the pH of the sodium sulfate solution at low temperatures, which favors the crystallization of new MKP crystals, and thereby the dissolution of the generated MKP crystals in the hardened body of MKPC mortar further decreases [15]. As shown in Figure 6b, the three M2 MKPC mortar samples also exhibited a distinct mass loss at approximately 100 °C, which is attributed to the loss of crystalline water in the MgKPO_4_·6H_2_O crystals. Additionally, the three M2 samples had an additional mass loss at approximately 620 °C, which is attributed to the thermal decomposition of CaCO_3_. Comparing the mass loss profiles of M0 and M2 samples before freeze–thawing at approximately 100 °C, the mass loss of the M2 samples (13.7%) was significantly lower than that of the M0 sample (16.38%), indicating that the lower MKP content produced in M2 is owing to the lower initial water–cement ratio of M2 and partial hydration reaction. After 200 freeze–thaw cycles in water, the mass loss of the M2 sample at approximately 100 °C (15.9%) increased, indicating an increase in the MKP content. After 200 cycles of freeze–thaw cycles in the sulfate solution, the mass loss of the M2 sample at approximately 100 °C (18.6%) further increased, indicating a further increase in the MKP content. The increased MKP content in the hardened bodies of the M2 specimens after 200 cycles of freeze–thawing in water and the sulfate solution was consistent with that of M0.

### 3.6. SEM EDS Analysis

**Figure 7:**
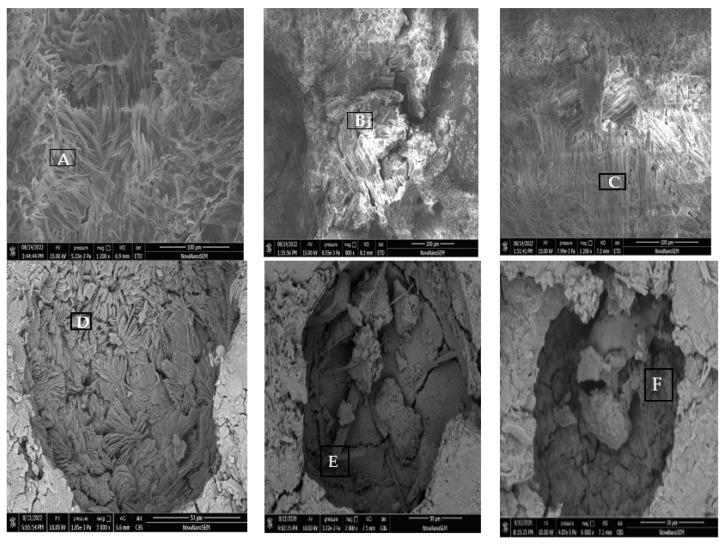
SEM micrographs of MKPC samples. (**A**) M0, before freeze–thaw cycling; (**B**) M0, after 200 freeze–thaw cycles in H_2_O; (**C**) M0, after 200 freeze–thaw cycles in 5% Na_2_SO_4_; (**D**) M2, before freeze–thaw cycling; (**E**) M2, after 200 freeze–thaw cycles in H_2_O; (**F**) M2, after 200 freeze–thaw cycles in 5% Na_2_SO_4_.

Figure 7A is the SEM image of the fracture surface of the M0 sample before freezing and thawing. The hydration products in the pores are mostly columnar and needle-shaped crystals. The hydration products are arranged in short-range order, and there are some gaps between the crystal clusters. According to the EDS results listed in Table 3, region A was mainly composed of O, Mg, P, and K elements, with a high content of O and Mg, and the molar ratio of P and K is similar. Therefore, based on the EDS and XRD results, region A is proposed to be MKP and unreacted MgO. The SEM micrograph of the M0 specimen after 200 freeze–thaw cycles in water (Figure 7B) shows that the filling of pores with hydration products in this specimen was significantly higher than that in Figure 7A, and the hydration products mainly consisted of short-range order needle-like crystals with a significantly higher degree of crystallization than that in Figure 7A. EDS analysis revealed that region B was composed of O, Mg, P, Cl, Na, and K, with the molar ratios of Mg, P, and K similar to that of MKP, indicating that the freeze–thaw cycling process was in progress (Table 3). This is the main reason for the slow strength loss of MKPC mortar specimens during the early stage of freeze–thaw cycling. Several cracks were observed on the walls of the air holes in the specimen shown in Figure 7B, caused by the compressive stress of ice expansion, which is one of the main reasons for the strength loss of specimens subjected to water freeze–thaw conditions. Figure 7C shows the SEM micrograph of the M0 specimen after 200 freeze–thaw cycles in 5% Na_2_SO_4_ solution. The filling of pores with hydration products in this specimen was higher compared with Figure 7A, and the hydration products mainly consisted of columnar crystals and amorphous phases, which confirms that the hydration reaction continued during the sulfate freeze–thaw cycling. Region C consisted of O, Mg, Na, Cl, P, K, and S, which may be identical to MKP. Notably, Na and Cl are derived from the composite inhibitor, and the presence of S confirms sulfate intercalation with the hydration products (Table 3).

As shown in Figure 7D, the pores of this specimen were significantly more filled with hydration products than those in Figure 7A, and the hydration products were composed of needle-like crystals and amorphous phases. Moreover, the structural density of M2 was significantly better than that in Figure 7A, which is one of the main reasons the initial strength of M2 was higher than that of specimen M0. EDS analysis shows that region D was mainly composed of O, Mg, P, and K, and the molar ratios of Mg, P, and K are similar, which may be attributed to MKP. As shown in Figure 7E, the filling of pores with hydration products remained high, and the size of the needle-like crystals of the hydration product was significantly larger than that in Figure 7D, indicating further hydration reactions under low-temperature water conditions and the presence of visible cracks in the pore walls, caused by ice expansion compressive stress, which is one of the main reasons for the strength loss of specimens subjected to water freeze–thaw cycling. Region E consisted of O, Mg, P, K, Ca, and Na, which may be attributed to MKP, but with intercalated Ca (Table 3). As shown in Figure 7F, the short columnar crystals in the pores of the specimen are closely arranged, which are relatively crystalline phases adhered to the surface of the crystals. Notably, region F consisted of O, Mg, Na, P, K, Ca, Cl, and S. The molar ratio of Mg, P, and K is similar to that of MKP, and the presence of S confirms that sulfate intercalation with the hydration products.

## 4. Conclusions and Prospect

The effects of mechanism limestone sand on the frost resistance of MKPC mortar were investigated. Based on the results obtained from the analysis and experimental works, the following conclusions can be drawn:

(1) The strength of three kinds of MKPC mortar specimens decreases gradually with the increase in freeze–thaw cycles. The strength of M0 and M1 is greater than 75% of the initial strength after 75 cycles of water freezing and thawing, and the strength of M2 is greater than 75% of the initial strength after 150 cycles of water freezing and thawing. After 200 cycles of water freezing and thawing, the flexural and compressive strength of M1 and M2 specimens still reached 21.3% and 23.0%, 26.6% and 32.2% of their initial strength, respectively. After 225 cycles of water freezing and thawing, the flexural and compressive strength of M3 specimens still reached 29.3% and 22.0% of their initial strength. When the sulfate freeze–thaw cycle reaches 75 times, the strength of M0 is lower than 75% of the initial strength. When the freeze–thaw cycle reaches 125 times, the strength of M1 is still higher than 75% of the initial strength. When the freeze–thaw cycle reaches 150 times, the strength of M2 is still higher than 75% of the initial strength. The flexural strength and compressive strength of M0 were 15.4% and 25.5% of the initial strength after 200 cycles of sulfate freezing and thawing, but M0 was destroyed after 225 cycles, and the flexural strength and compressive strength of M1 and M2 were 44.1% and 49.3%, 43.5% and 45.7% of the initial strength, respectively. The results show that the resistance to freezing and thawing of M3 specimens made of common river sand and limestone sand is obviously higher than that of MKPC mortar (M0 and M1) made of single river sand and limestone sand. The resistance of M0 to sulfate freezing and thawing is lower than that of water freezing and thawing, but the resistance of M1 and M2 containing limestone sand is obviously higher than that of M0 mortar containing river sand alone.
(2) With the increase in freeze–thaw cycles, the mass change rate of M0–M2 specimen increases at first and then decreases gradually. When water is frozen and thawed 225 times, the mass loss rates of M0, M1, and M2 specimens are 0.37%, 0.31%, and 0.08%, respectively. When the sulfate solution is frozen and thawed 225 times, the mass loss rates of M0, M1, and M2 specimens are 0.31%, 0.18%, and 0.02%, respectively, which is slightly lower than that of the water frozen and thawed for 225 times. The results show that the mass loss of MKPC mortar is still far lower than the 5% damage standard when it is frozen and thawed 225 times in water and 5% Na_2_SO_4_ solution.
(3) The initial water absorption of M0–M2 specimens is 0.64%, 0.51%, and 0.67%, respectively (before freezing and thawing), which indicates that the initial open porosity of M2 specimens is obviously lower than that of M0 and M1 specimens, which should be attributed to the decrease in water–cement ratio of MKPC mortar with the same fluidity due to the reasonable mix of two aggregates. With the increase in freeze–thaw cycles, the water absorption of the three MKPC mortar specimens gradually increased. After 225 water freeze–thaw cycles, the water absorption was 2.98%, 3.15%, and 2.20%, respectively. After 225 sulfate freeze–thaw cycles, the water absorption of M0–M2 specimens is 2.58%, 2.46%, and 1.83% respectively, which is lower than that of the corresponding specimen after 225 sulfate freeze–thaw cycles in water.

This study confirms that limestone sand can significantly improve the sulfate freezing and thawing resistance of MKPC mortar. According to the existing research results, it is inferred that under the low-temperature environment, the active calcium carbonate on the surface of limestone sand will react with the sulfate in the medium to generate a new phase to fill the pores of the hardened body. However, no new phase is found in the XRD analysis of this study, which needs to be verified by the subsequent test research.

## Figures and Tables

**Table 1:** The physical properties of fine aggregates.

Category	Fineness Modulus	Clay Content (%)	Bulk Density (kg/m^3^)	Gradation
Common river sand	2.53	0.80	1450	JGJ52-2006, Zone II [20]
Limestone Sand	3.65	0.00	1460	JGJ52-2006, Zone II [20]

**Table 2:** Some mechanical properties of MKPC mortar.

Code	Fine Aggregate Variety	Ww/WMKPC	Fluidity (mm)	Flexural Strength (MPa) 3d 28d		Compressive Strength (MPa) 3d 28d	
M0	100%Rs	0.20	140	6.16	8.02	35.1	51.6
M1	100%Ls	0.20	141	6.46	8.32	35.2	50.3
M2	34%Rs + 66%Ls	0.18	138	7.32	8.25	39.3	54.8

Note: Reference to ASTM C1437-2015 “Standard Test Method for Flow of Hydraulic Cement Mortar” for test methods. RS refers to common river sand, LS refers to limestone sand, and W refers to water.

**Table 3:** Distribution of elements in the region of MKPC mortar samples.

Element Name		O	Mg	S	P	Cl	K	Ca	Na
Percentage of atoms (%)	A	71.63	9.74		9.52		9.11		
	B	69.17	10.83		9.65	0.14	9.18		1.03
	C	69.21	8.84	1.18	9.08	0.24	9.30		2.15
	D	72.30	10.36		8.12		9.22		
	E	73.52	8.43		6.95		6.07	2.11	2.92
	F	66.35	7.47	2.56	8.85	0.35	9.55	2.86	2.01

## Data Availability

Not applicable.
